# Seed yield as a function of cytokinin-regulated gene expression in wild Kentucky bluegrass (*Poa pratensis*)

**DOI:** 10.1186/s12870-024-05421-w

**Published:** 2024-07-20

**Authors:** Jinqing Zhang, Xue Ha, Huiling Ma

**Affiliations:** 1 College of Forestry and Prataculture, Ningxia University, Yinchuan, 750021 China; 2 College of Pratacultural Science, Key Laboratory of Grassland Ecosystem, Pratacultural Engineering Laboratory of Gansu Province, Gansu Agricultural University, Ministry of Education, Sino-U.S. Center for Grazingland Ecosystem Sustainability, Yingmencun, Anning District, Lanzhou, Gansu 730070 China

**Keywords:** Wild Kentucky bluegrass, Cytokinin, Gene expression patterns, Seed yield parameters, Nutrient

## Abstract

**Background:**

Kentucky bluegrass (*Poa pratensis* L.) panicle development is a coordinated process of cell proliferation and differentiation with distinctive phases and architectural changes that are pivotal to determine seed yield. Cytokinin (CK) is a key factor in determining seed yield that might underpin the second “Green Revolution”. However, whether there is a difference between endogenous CK content and seed yields of Kentucky bluegrass, and how CK-related genes are expressed to affect enzyme regulation and downstream seed yield in Kentucky bluegrass remains enigmatic.

**Results:**

In order to establish a potential link between CK regulation and seed yield, we dissected and characterized the Kentucky bluegrass young panicle, and determined the changes in nutrients, 6 types of endogenous CKs, and 16 genes involved in biosynthesis, activation, inactivation, re-activation and degradation of CKs during young panicle differentiation of Kentucky bluegrass. We found that high seed yield material had more meristems compared to low seed yield material. Additionally, it was found that seed-setting rate (SSR) and lipase activity at the stage of spikelet and floret primordium differentiation (S3), as well as 1000-grain weight (TGW) and zeatin-riboside (ZR) content at the stages of first bract primordium differentiation (S1) and branch primordium differentiation (S2) showed a significantly positive correlation in the two materials. And zeatin, ZR, dihydrozeatin riboside, isopentenyl adenosine and isopentenyl adenosine riboside contents were higher in seed high yield material than those in seed low yield material at S3 stage. Furthermore, the expressions of *PpITP3*, *PpITP5*, *PpITP8* and *PpLOG1* were positively correlated with seed yield, while the expressions of *PpCKX2*, *PpCKX5* and *PpCKX7* were negatively correlated with seed yield in Kentucky bluegrass.

**Conclusions:**

Overall, our study established a relationship between CK and seed yield in Kentucky bluegrass. Perhaps we can increase SSR and TGW by increasing lipase activity and ZR content. Of course, using modern gene editing techniques to manipulate CK related genes such as *PpITP3/5/8*, *PpLOG1* and *PpCKX2/5/7*, will be a more direct and effective method in Kentucky bluegrass, which requires further trial validation.

**Supplementary Information:**

The online version contains supplementary material available at 10.1186/s12870-024-05421-w.

## Background

Kentucky bluegrass (*Poa pratensis* L.) is an important component in grassland and meadow vegetation that is mainly distributed in cold and humid regions in boreal and north temperate zones [1], so it can serve as excellent forage and lawn grass. Additionally, Kentucky bluegrass also has important ecological applications [2]. Furthermore, Kentucky bluegrass is also a natural facultative apomictic species, which can stably inherit genetic material adapted to the environment and keep generation variation [3]. Therefore, Kentucky bluegrass exhibits diverse reproductive modes including sexuality and apomixis, making it more difficult to study its seed related trait and cultivate high-yield seed varieties. Kentucky bluegrass has an indeterminate inflorescence, which develops from panicle differentiation. During its panicle morphogenesis, the multiple inflorescence meristems that form and give rise to spikelets and florets, which are pivotal determinants of seed yield [4, 5]. During panicle differentiation, the sink-source relationship changes, and photosynthetic products are distributed from vegetative organs to flowers and seeds [6]. Specifically, these photosynthetic products include sugars, proteins and other nutrients that are crucial to the successful development of the panicle [7]. Ultimately, these changes during panicle differentiation will affect the number of spikelets, florets, and seeds that directly affect seed yield, as well as the Kentucky bluegrass’ economic benefits [8]. The developmental process of panicle can be influenced by genetic factors, environmental conditions and cultivation practices [9]. Thus, we need to focus on the pattern of panicle differentiation and development to improve cultivation practices to obtain high-quality and high-yield seeds of grass.

Phytohormones are products of plant metabolic reactions, and are known to impact panicle growth and development [10–12]. In particular, cytokinin (CK) plays an important role in panicle differentiation, and is degraded by CK oxidizes/dehydrogenase (CKX) to adenine or adenosine [13–15]. Interestingly, plants that produce higher mass seeds appear to sustain peak CK content at higher levels and for longer time [16]. Previously, Ashikari et al. (Ashikari et al., 2005) have indicated that there is a positive relationship between the number of seeds, branches, and CK content in rice (*Oryza sativa*). Other previous reports have suggested that modern genome editing tools could be employed to target and manipulate CK levels to increase seed yield [13]. In summary, CK is a key factor in determining seed yield and might underpin the second “Green Revolution” [17]. The bioactive CK concentration is regulated by several multigene families, including isopentenyl transferases (*IPT*) for CK biosynthesis, Lonely Guy (*LOG*) for activation, zeatin O-glucosyltransferases (*ZOG*) for reversible inactivation, β-glucosidases (*GLU*) for reactivation and CK oxidase/dehydrogenase (*CKX*) for degradation [18]. For example, downregulation or deletion of the *OsCKX* gene leads to altered inflorescence meristem and rachis branch organization [19]. Additionally, branching differentiation is strongly inhibited in *LOG* mutants, and only one floret forms at the top of each branch stem [20]. In barley (*Hordeum vulgare*), silencing of the *HvCKX1* gene and subsequent elevation of CK levels have increased the number of seeds per plant, the weight of kernel as well as total seed yield [21, 22]. Moderate down-regulation of *GhCKX* gene expression can prevent CK degradation in cotton (*Gossypium herbaceum*) and lead to increase of fiber and seed yield [23]. Different transgenic strategy, using autoregulatory senescence inhibition system based on elevation of CK levels through PSAG12-driven expression of CK biosynthetic gene *IPT*, has been used to delay leaf senescence without abnormalities in growth and development, and increase in grain yield and biomass, dramatic improvement in horticultural performance, and/or enhanced tolerance to drought stress in many crops [24]. Taken together, the CK status and its regulation by *CKXs* is a well-established determinant of seed yield in many crops, so precise control of CK levels could pave the way of CK-derived compounds among the commercially successful agrochemicals.

Overall, abnormal CK homeostasis that is activated by senescence-related metabolisms lead to panicle primordia absent and their typical meristem characteristics. Currently, reports of CK regulation of seed yield are not limited to model plants and major grass grain crops, and have also been reported in allotetraploid rape (*Brassica napus* L.) [25]. However, there have been no reports on the application of CK in regulating seed yield of *Poa pratensis.* Thus, understanding CK and its regulation by genes in Kentucky bluegrass could lead to agriculturally relevant applications for plant productivity. Here, we used wild germplasm materials of Kentucky bluegrass from Gannan (GN) and Longnan (LN), Gansu Province of China, to trace the morphological changes during the transition from the vegetative to the reproductive phase of development, and to extend and clarify the knowledge of the initiation, organization, and early inflorescence development. Our previous study found, between GN and LN, the seed yield-related traits were differed significantly, in which the single seed yield for GN was 1.91 g, while that in LN was only 1.02 g [26]. We also measured nutrients, multiple endogenous CKs, and expression levels of 16 CK-related genes during panicle differentiation to confirm their effects on seed yield parameters. The results found that the CK regulatory pathway during panicle differentiation had an effect on seed production, and established a potential link between CK level and the seed yield parameters. Ultimately, we were able to define the relationships between CK-related genes and their corresponding enzymatic regulation that affect seed yield and quality during panicle differentiation in wild germplasm materials of Kentucky bluegrass. Together, these genetic regulatory factors of CKs can be used to improve seed yield parameters of other plant species to improve crop-yields under agronomically-relevant field conditions in the future.

## Results

### Statistical comparison of seed yield traits in Kentucky bluegrass

**Fig. 1:**
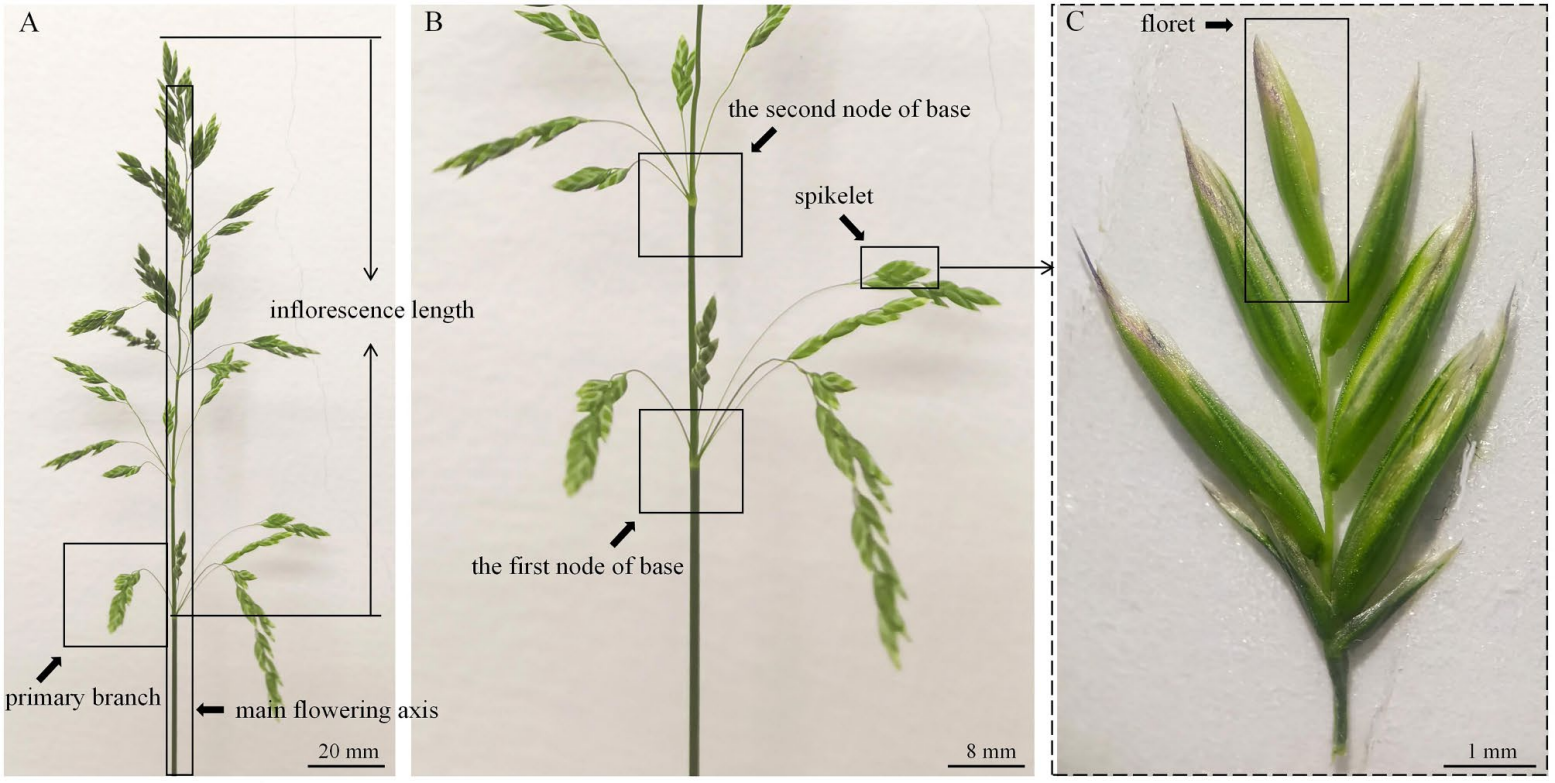
The inflorescence structure of wild germplasm materials of Kentucky bluegrass. (**a**) entire inflorescence, main flowering axis, and primary branch were shown. (**b**) the first and second node of the base, and spikelet were shown. (**c**) the floret was shown. According to the inflorescence structure, we defined the seed yield-related traits

**Fig. 2:**
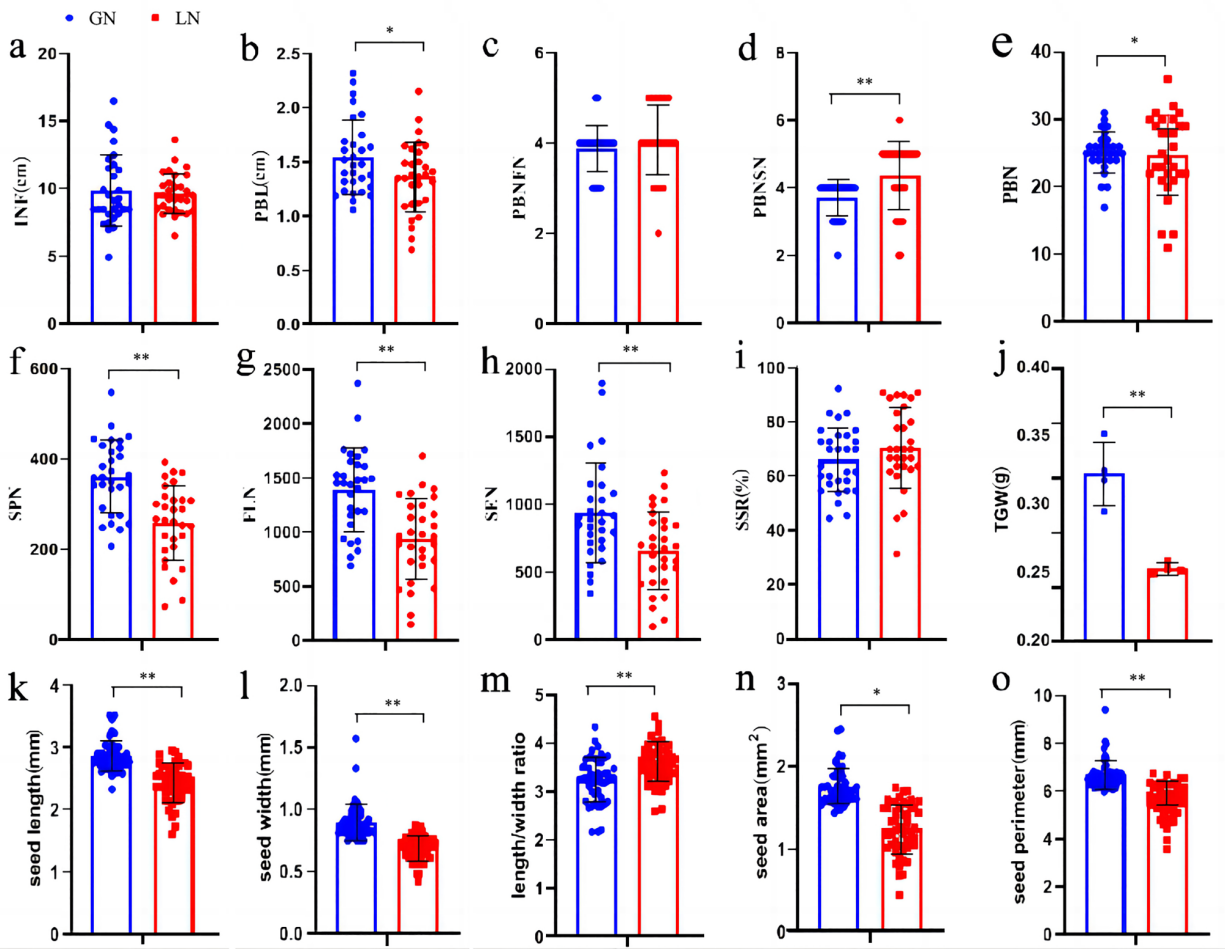
Statistical comparison of different seed yield-related traits in Kentucky bluegrass. (**a**) INF: inflorescence length. (**b**) PBL: primary branch length. (**c**) PBNFN: primary branch number at the first node of base on a main flowering axis. (**d**) PBNSN: primary branch number at the second node of base on a main flowering axis. (**e**) PBN: primary branch number on a main flowering axis. (**f**) SPN: spikelet number on a main flowering axis. (**g**) FLN: floret number on a main flowering axis. (**h**) SEN: seed number on a main flowering axis. (**i**) SSR: seed-setting rate on an inflorescence. (**j**) TGW: 1000-grain weight. (**k**) seed length. (**l**) seed width. (**m**) seed length/width ratio. (**n**) seed area. (**o**) seed perimeter. Among them, TGW has four biological replicates. ** indicates extremely significant differences at the *P* < 0.01 level between two materials, and * indicates significant differences at the *P* < 0.05 level, according to *t-tests*. Bars represent the standard error

We first characterized several yield-related traits in Kentucky bluegrass that are illustrated in Fig. 1 and described in Table 1. The statistical comparison of these seed yield-related traits in GN (blue) and LN (red) is presented in Fig. 2. Between the inflorescence length (INF), primary branch number at the first node of the base on a main flowering axis (PBNFN), primary branch number on a main flowering axis (PBN) and seed-setting rate on an inflorescence (SSR) (Fig. 2a, c, e, and i), there were no differences between the two materials (*P* > 0.05). Additionally, we found that only primary branch number at the second node of base on a main flowering axis (PBNSN) in LN was significantly higher than that in GN (Fig. 2d), while the remaining traits were higher in GN (*P* < 0.05), and except for primary branch length (PBL) (Fig. 2b), the spikelet number on a main flowering axis (SPN), floret number on a main flowering axis (FLN), seed number on a main flowering axis (SEN) and 1000-grain weight (TGW) (Fig. 2f, g, h and j) all reached significant levels (*P* < 0.01). The seed growth parameters were also extremely significantly lower in LN, except for length/width ratio in GN (Fig. 2m). The seed length, width, area and perimeter in GN were all significantly higher than that in LN (Fig. 2k, l and n and o).

**Table 1:** The characteristic and their abbreviation of seed yield parameters

Characteristic	Abbreviation
inflorescence length	INF
primary branch number on a main flowering axis	PBN
primary branch number at the first node of the base on a main flowering axis	PBNFN
primary branch number at the second node of base on a main flowering axis	PBNSN
primary branch length	PBL
spikelet number on a main flowering axis	SPN
floret number on a main flowering axis	FLN
seed number on a main flowering axis	SEN
seed-setting rate on an inflorescence	SSR
1000-grain weight	TGW

### Dynamic process of panicle differentiation and development

Panicle differentiation comprised a set of sequential and developmental events featured by transitions from the early inflorescence stage to primary branch, then to secondary branch, followed by spikelet and then the final floret. According to the different morphological characteristics of growth and differentiation at each stage, it could be divided into five stages.

**Fig. 3:**
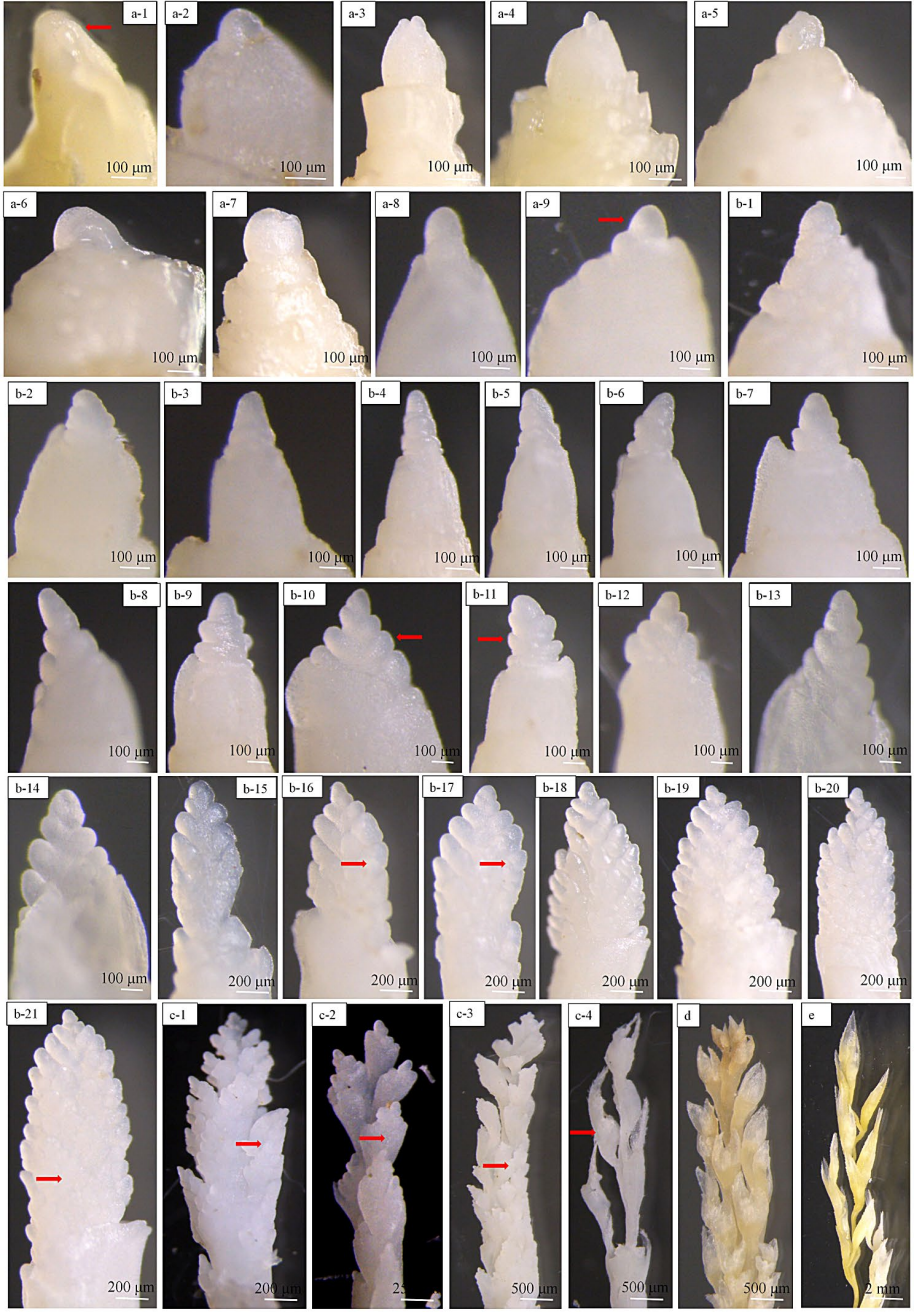
Dynamic characteristics observation of the panicle differentiation in Kentucky bluegrass. (**a**1—**a**9) first bract primordium differentiation; (**b**1—**b**21) branch primordium differentiation; (**c**1—**c**4) spikelet and floret primordium differentiation; (**d**) stamen and pistil formation; (**e**) stamen and pistil development. Red arrows indicate signature changes in the morphological characteristics of young panicle

**Fig. 4:**
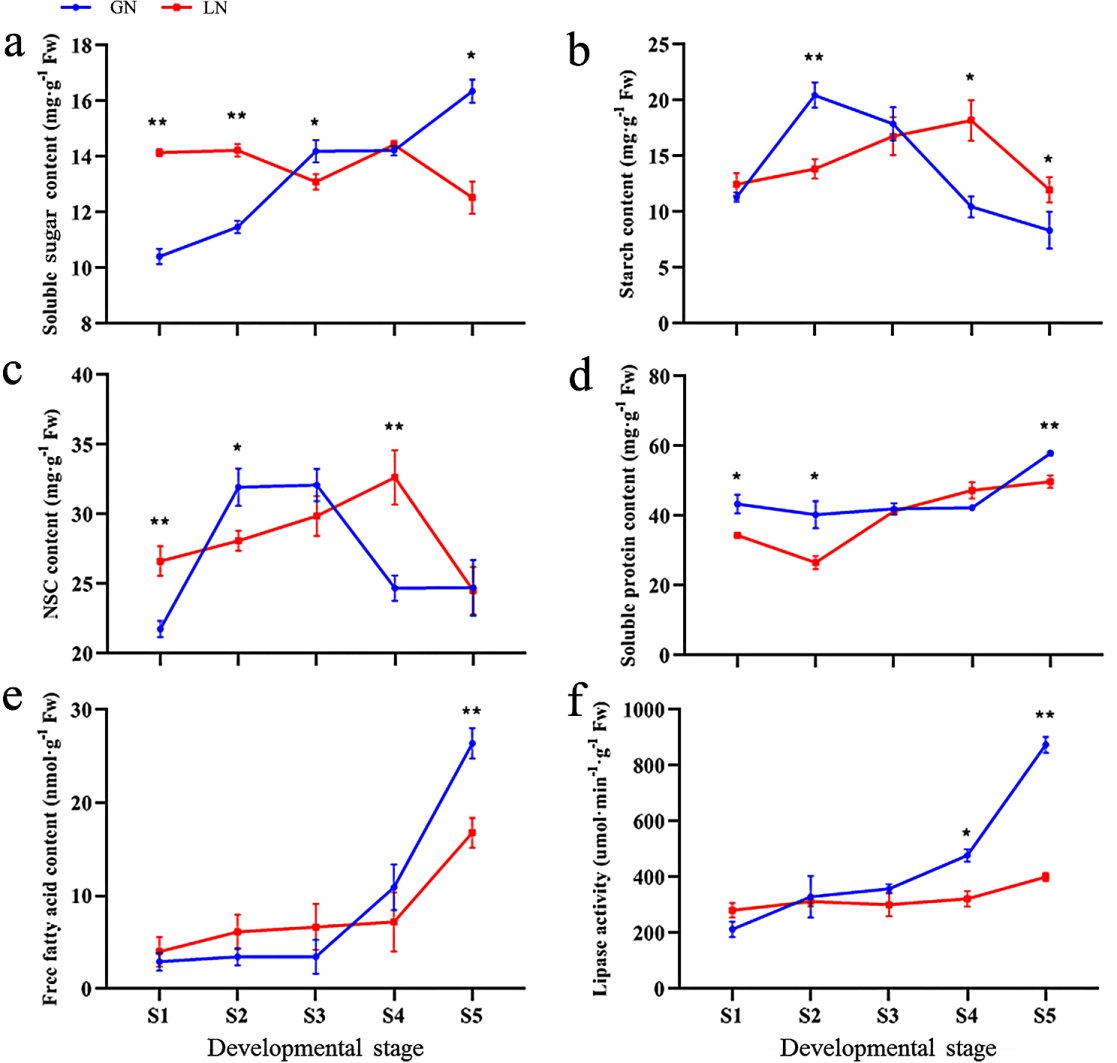
Changes of nutrients during the panicle differentiation in Kentucky bluegrass. (**a**-**e**) The contents of soluble sugar, starch, NSC, soluble protein and free fatty acid; (**f**) The lipase activity. The red lines represent material LN, and the blue lines represent material GN. ** indicates extremely significant differences at the *P* < 0.01 level between two materials at same stage, and * indicates significant differences at the *P* < 0.05 level, according to *t-tests*. Bars represent the standard error

**Fig. 5:**
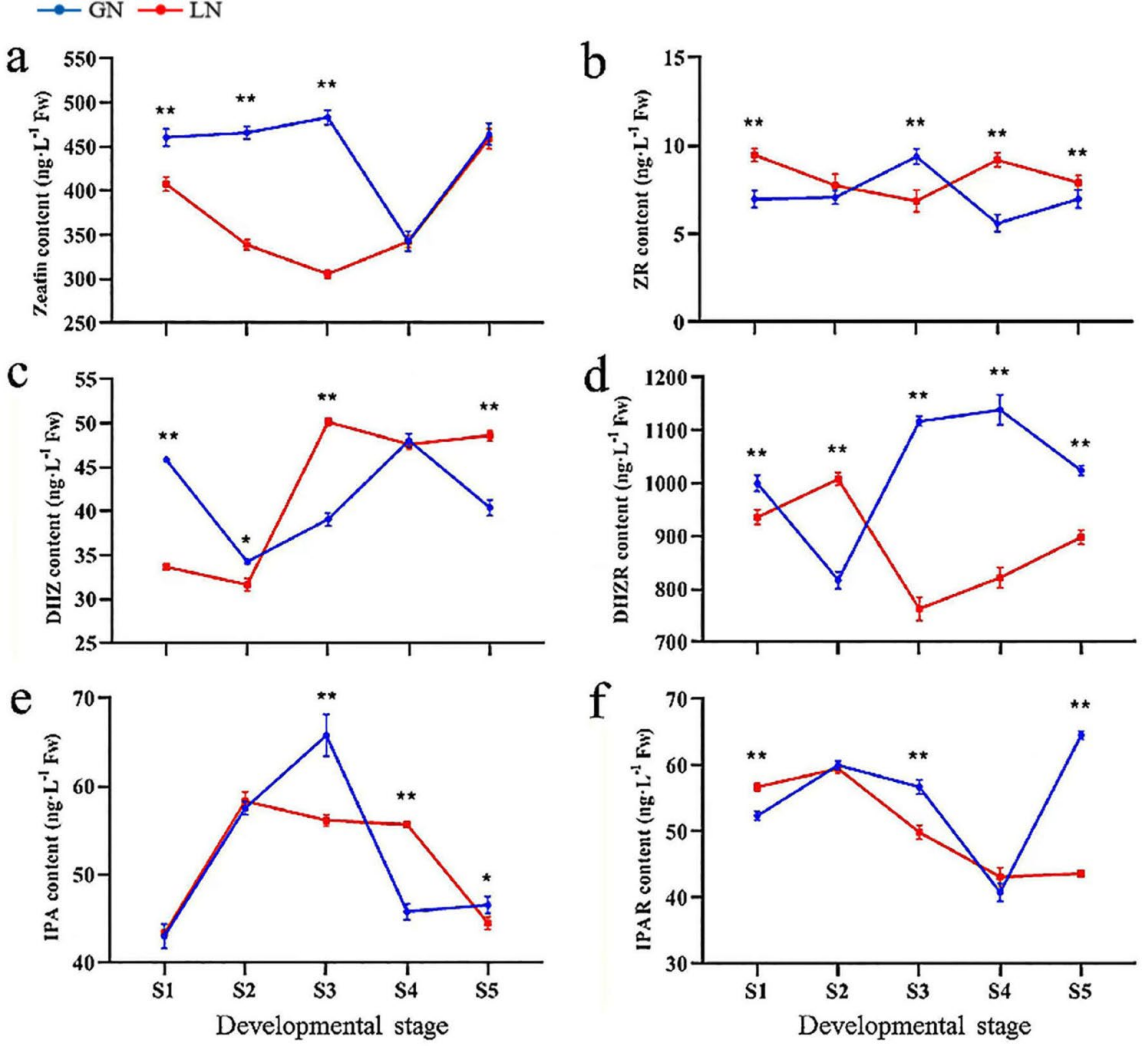
Changes of 6 types of major transport forms and active forms of CK contents during the panicle differentiation in Kentucky bluegrass. (**a**-**f**) The contents of zeatin, ZR, DHZ, DHZR, IPA and IPAR, respectively. ZR: zeatin-riboside; DHZ: dihydrozeatin; DHZR: dihydrozeatin riboside; IPA: isopentenyl adenosine; IPAR: isopentenyl adenosine riboside. The red lines represent material LN, and the blue lines represent material GN. ** indicates extremely significant differences at the *P* < 0.01 level between two materials at same stage, and * indicates significant differences at the *P* < 0.05 level, according to *t-tests*. Bars represent the standard error

1. First bract primordium differentiation (S1): When the young panicles began to differentiate, a small protrusion first appeared at the base of the growth point at the top of the shoot tip and inside the parietal primordium, which was the first bract primordium (Fig. 3a-1—Fig. 3a-4). The differentiation of the first bract primordia was the symbolic starting point of reproductive growth. Subsequently, the growth tip gradually increased, and the length exceeded the width and forms a cone (Fig. 3a-5—Fig. 3a-9).

**Fig. 6:**
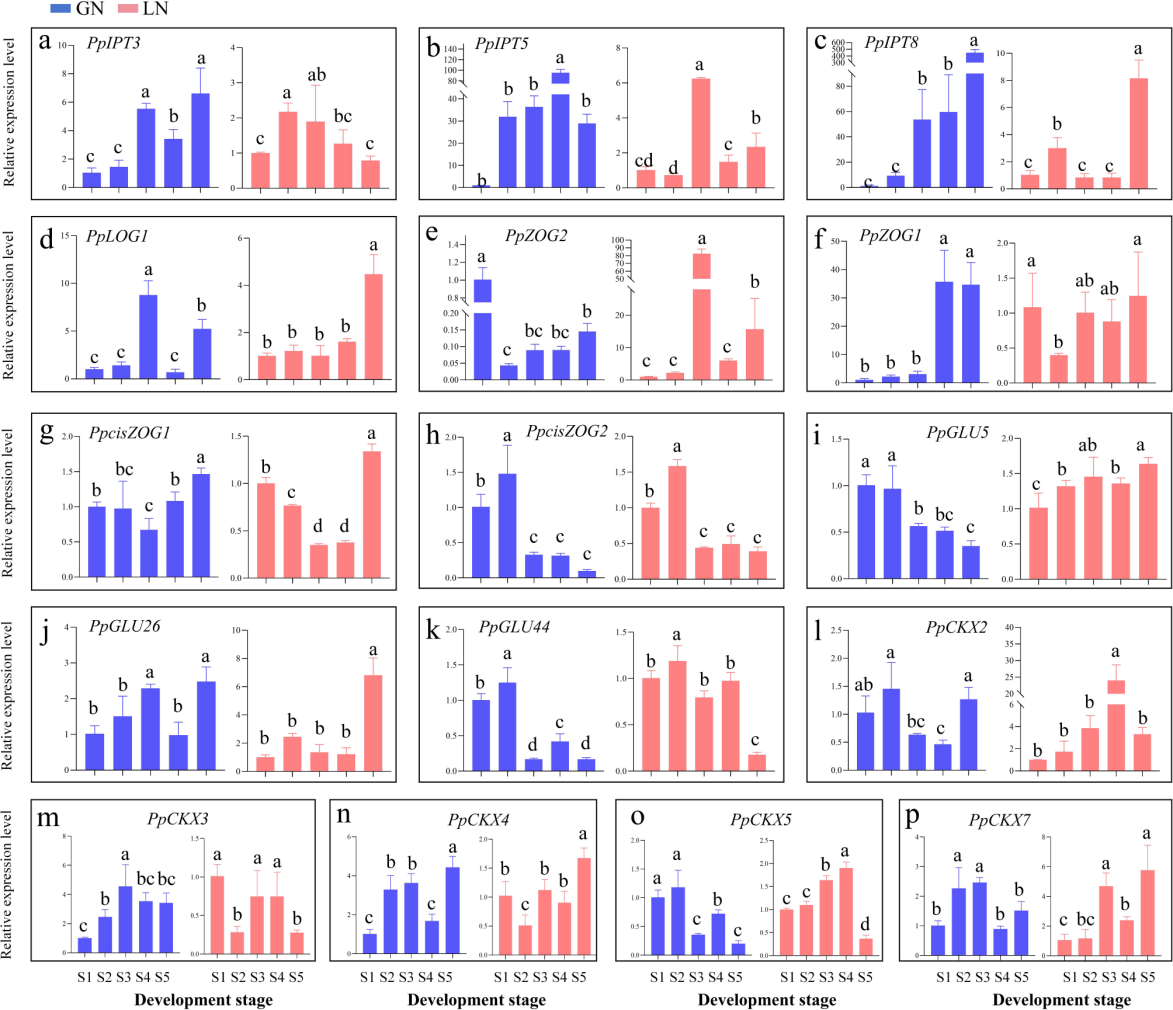
The qRT-PCR validation of CK-related genes at different developmental stages of panicle in two materials. Using S1 as the control, the relative expression level of the other four stages were calculated by 2^−∆∆Ct^ in GN and LN, respectively. Three *PpIPT* genes (**a**-**c**) for CK biosynthesis, a *PpLOG* gene (**d**) associated with CK activation, four *PpZOG* genes (**e**-**h**) for CK inactivation, three *PpGLU* genes (**i**-**k**) for CK re-activation, and five *PpCKX* genes (**l**-**p**) for CK degradation. The red columns represent material LN, and the blue columns represent material GN. Different letters indicate statistical significance, where the same letter indicates no significant difference between different developmental stages, according to one-way ANOVA test (*P* < 0.05). Bars represent the standard error

**Fig. 7:**
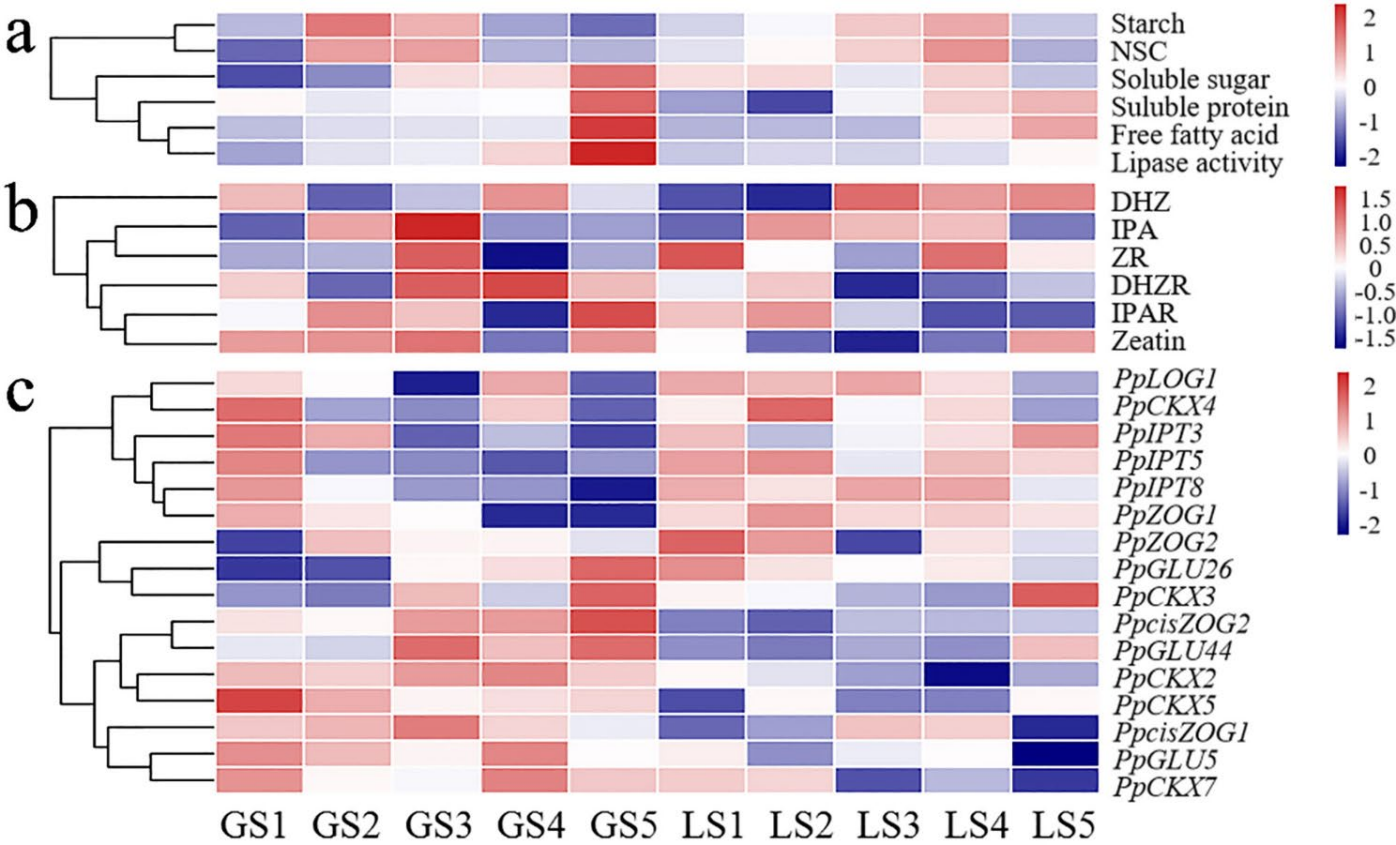
Comprehensive trait heatmap for the different nutrients, CKs, and CK-related genes analyzed in the GN and LN. Red indicates the higher content or gene expression level, and blue indicates the lower content or gene expression level. The darker color indicates the value is the higher. (**a**) nutrient content; (**b**) endogenous CKs contents; (**c**) expression levels of the CK-related genes were calculated by ∆Ct

**Fig. 8:**
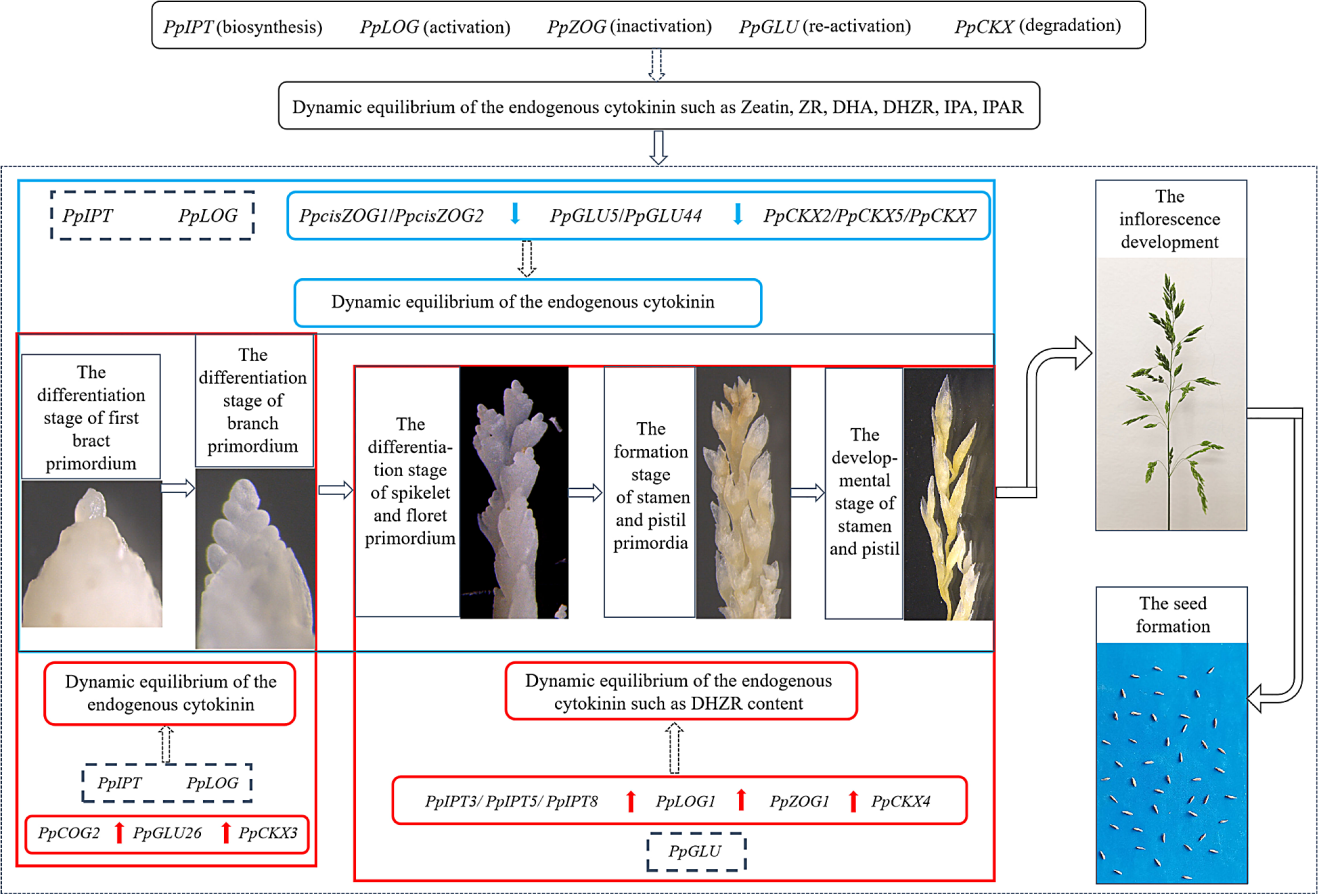
A hypothetical molecular regulatory network that produces high seed yield parameters in GN. The modules colored in red are highly expressed and modules colored in green are lowly-expressed. The real boxes indicate the CK-related genes analyzed the dotted boxes indicate the potential CK-related genes that may be highly- or under-expressed

2) Branch primordium differentiation (S2): The first bract primordium continued to increase to form a ring, and the growth cone differentiated into the second and third bract successively (Fig. 3b-1—Figs. 3b-5). The axil of the first bract primordium then protruded outward to produce new protrusions, which were the primary branch primordia (Fig. 3b-1—Fig. 3b-5). Protrusions formed from the bottom to the top, and finally reached the top of the growth cone, which marked the completion of the primary branch (Fig. 3b-14). The primary branch primordium near the lower part of the growth cone grew the fastest where the base first formed a secondary branch primordium and gradually differentiates upwards (Fig. 3b-15, 16). When the primary branch primordium at the top differentiated into the secondary branch primordia, the secondary branch primordium in the lower part of the growth cone produced new protrusions that further grow and differentiate to form tertiary branch primordia (Fig. 3b-17—Fig. 3b-21).
3) Spikelet and floret primordium differentiation (S3): After the tertiary branch primordium was formed, protrusions were first produced at the base of the branch primordium, and then differentiated into spikelet primordium (Fig. 3c-1). The glume primordium then differentiated in the axils of the spikelet primordium where the lemma and palea primordia differentiated in the axil of the glume primordium. Subsequently, the floret primordium differentiated in the axils of the palea primordia (Fig. 3c-2). The lemma, palea, and floret primordia of the second floret then gradually differentiated continually, until the final floret primordium was formed (Figs. 3c-3, 4).
4) Stamen and pistil primordial formation (S4): After floret primordium formation, the stamen and pistil primordia began to appear. After, the stamen primordium developed into filaments and anthers, and the pistil primordium differentiated into the ovary, style, and stigma. In this process, the stamens primordium grew faster than the pistil primordium. Meanwhile, the glume developed quickly, and the lemma and palea developed slower in comparison. When the lemma and palea completely surrounded the stamen and pistil, the morphology of the young panicle had been established (Fig. 3d).
5) Stamen and pistil development (S5): After the stamen and pistil differentiated, they further developed in preparation for double fertilization. The stamen underwent microsporogenesis and male gametophyte development, while the pistil underwent macrosporogenesis and female gametophyte development that eventually formed egg and sperm cells for double fertilization. During this process, Kentucky bluegrass panicles continued to grow and develop until they formed mature panicle that completed the entire inflorescence (Fig. 3e).

The panicle length, phenophase (The times of plant growth, development, activity, and the response of biological changes to climate are called phenophase) and date corresponding to each stage from panicles from LN and GN were shown in Table 2. In addition, the corresponding relationship between panicle length, phenophase and each developmental stage was similar. However, the collection dates for samples from GN were 12 days later than those collected from LN. Furthermore, with special attention to the number and size of meristems in the two materials, it was found that GN has more meristems compared to LN (Table 2). One point to be explained was that subsequent determination of nutrition, several endogenous CK, and expression level of CK-related genes may be affected due to the inconsistent sampling date of the two materials. However, we ensured the consistency of the growth environment and the developmental stage before and after sampling; In addition, we collected five consecutive time points, which could also reduce the errors caused by inconsistent sampling dates.

**Table 2:** The panicle length and phenophase corresponding to developmental stage

Stage of differentiation and development	Phenophase	LN			GN		
		Panicle length (mm)	Date (2022)	Number of meristems	Panicle length (mm)	Date (2022)	Number of meristems
The differentiation stage of first bract primordium	regreening stage	0.05—0.15	3.1—3.5	1—2	0.08—0.20	3.13—3.17	1—2
The differentiation stage of branch primordium	regreening stage, tillering stage	0.15—1.35	3.6—3.18	2—26	0.20—1.52	3.18—4.30	2—33
The differentiation stage of spikelet and floret primordia	jointing stage	1.35—2.00	3.19—3.25	26—30	1.52—2.60	4.1—4.7	33—36
The formation stage of stamen and pistil primordia	booting stage	2.00—10.00	3.26—4.10	30—32	2.60—13.00	4.8—4.22	36—41
The developmental stage of stamen and pistil	booting stage, heading stage	10.00—150.00	4.11—4.25	32—35	13.00—185.00	4.23—5.7	41—46

### Changes of nutrient content during panicle differentiation

Sugars, proteins and fats were the main nutrients during panicle development, and they were the bases for successful panicle differentiation and seed production in Kentucky bluegrass. To better understand nutrient content, we determined the contents for the soluble sugar, starch, soluble protein, free fatty acid and lipase activity for the panicle at five different developmental stages (Fig. 4). Overall, the nutrients were mostly different for panicles from the GN and LN. The soluble sugar content from LN was higher at early panicle differentiation stages (S1 and S2), but was higher in GN at the S3 and S5 stages (Fig. 4a). The starch content in LN was lower at S1 stage, and higher at the S4 and S5 stages (Fig. 4b), while the nonstructured carbohydrates (NSC) content in LN was higher at S1 and S4 stages (Figs. 3c and 4b). Additionally, the soluble protein content in GN was substantially higher than that of LN (Fig. 4d). However, free fatty acid content and lipase activity were similar in the early developmental stages (S1-S4), but significantly increased in GN in the S5 stages (Fig. 4e, f).

### Endogenous CKs changes during panicle differentiation

After exploring nutrient changes in the panicle, we selected 6 types of CKs in major transport and active forms to determine whether the changed endogenous CKs in response to panicle differentiation. These CKs included zeatin, zeatin-riboside (ZR), dihydrozeatin (DHZ), dihydrozeatin riboside (DHZR), isopentenyl adenosine (IPA) and isopentenyl adenosine riboside (IPAR), and their changes in GN and LN during each panicle developmental stage were shown in Fig. 5. In general, we found the CKs were dynamically changing based on the two materials and the stage of young panicle development. The zeatin content in GN was also significantly higher than that in LN at S1, S2 and S3 (*P* < 0.01), and there was no difference between the two materials in the latter two stages (Fig. 5a). Except for S3, the ZR content in LN was higher than that in GN at the other stages (Fig. 5b). In the first two stages, the DHZ content in GN was also higher than that in LN, and significantly lower at S3 and S5 (Fig. 5c). For DHZR content, except for S2, it was significantly higher in GN than that in LN at other stages (Fig. 5d). There was not any differences in IPA content between the two materials at S1 and S2, while it was higher in GN than that in LN at S3 and S5, and significantly lower at the S4 stage (Fig. 5e). Further, there was not any differences in IPAR content between two materials at S2 and S4, while it was significantly higher in GN than that in LN at S3 and S5, and significantly lower at S1 (Fig. 5f). Overall, these variation patterns in the 6 types of CKs were not obvious and the trends were different in the two materials by the different developmental stages. Interestingly, it was found that out of the six CK concentrations quantified in this study, five (zeatin, ZR, DHZR, IPA and IPAR) kinds were higher in the GN than LN at S3 (Fig. 5).

To determine the relationship between seed yield-related traits and nutrients, CKs contents respectively, correlation analysis was performed (Table S1, S2, S3 and S4). By comparing the differences between the two materials, we screened for nutrient indicators and endogenous CK that showed consistent correlation with seed yield parameters in both materials (Table 3). Our results found that SEN and NSC at S3, as well as SSR and lipase activity at the S3 stage showed significant positive correlation (Table 3, Table S1 and Table S2). Additionally, we found that PBNSN and ZR content at the S1 and S2 stages showed significantly negative correlation in two materials. Nevertheless, the TGW and ZR content at the S1 and S2 stages showed significantly positive correlation in the two materials (Table 3, Table S3 and Table S4).

**Table 3:** The nutritional index and endogenous CK were correlated with the parameters of Kentucky bluegrass seed yield

Term	Developmental stage	SEN		SSR		PBNSN		TGW	
		GN	LN	GN	LN	GN	LN	GN	LN
NSC	S3	0.998*	1.000**						
lipase activity	S3			0.999*	0.999*				
ZR	S1					-1.000**	-1.000**	1.000**	1.000**
	S2					-1.000**	-1.000**	1.000**	1.000**

Note: NSC: Nonstructured carbohydrates; ZR: Zeatin-riboside; SEN: seed number on a main flowering axis; SSR: seed-setting rate on an inflorescence; PBNSN: primary branch number at the second node of base on a main flowering axis; TGW: 1000-grain weight

### Changes in expression levels of CK-related genes during panicle differentiation

The independent and variable expression profiles of different CK regulatory gene families provided useful flexibility for independently manipulating the endogenous bioactive CK levels in reproductive tissues or organs to achieve maximal seed yield. Therefore, 16 CK-related genes were selected to determine their expression levels and verify the relationship between gene expression patterns in the panicle and seed yield for Kentucky bluegrass (Table 4, Table S5 and Table S6). In order to better analyze the changing trends of each gene in different materials and clarify the up and down regulation trends of genes when comparing two materials at the same period, we used different samples as controls to calculate the relative expression levels of genes, which can provide a more comprehensive understanding of the expression of CK related genes (Fig. 6 and Fig. S1).

**Table 4:** Primer sequences used for qRT-PCR analysis of CK-related genes

Work Name	Description	Forward primer	Reverse primert
*IPT3*	adenylate isopentenyltransferase 3	GATGCTGGAGTGGTACACGG	CGTCTAACTCAGCACAAGCC
*IPT5*	adenylate isopentenyltransferase 5	ACGAGATCAAGGCCAACACG	GAGGAATACCTCCGTGGCATC
*IPT8*	adenylate isopentenyltransferase 8	CATAGGCGTCCAAGATCCCC	GTCGACAGATCGCCGTATGA
*LOG1*	cytokinin riboside 5’-monophosphate phosphoribohydrolase	TGCCTTTATAGCGCTCCCTG	CAGAGCGTTGTAGTAGCCGT
*COG2*	cytokinin-O-glucosyltransferase 2	CGTGTACGTGACCTTCGTGA	GGAATCGTCTCGAACCGGAA
*ZOG1*	zeatin O-glucosyltransferase 1	TGACGAGTGTTGACGTGGAG	ATGTCCTCCAGGTCGGTGTA
*cisZOG1*	cis-zeatin O-glucosyltransferase 1-like	CAATATCGTGGCCTTCCCCT	TACCCGCGATAAACGACGAC
*cisZOG2*	cis-zeatin O-glucosyltransferase 2-like	GAACTCGCTGCTCGAAGGAC	ATTGCCCGCCTGATAACCTC
*GLU5*	beta-glucosidase 5	TCCAGGAGAATGGCAAGTCG	CCTGTCGGTATCGTCGAGAG
*GLU26*	beta-glucosidase 26	ACGCCTACCGGTTTTCCATC	AGTGTCAGCGGGAGGTCATA
*GLU44*	beta-glucosidase 44	ACTACCTGCTTCAGAAAGGCA	AGTGCTTTACTCGATCGCCA
*CKX2*	cytokinin dehydrogenase 2	CTCTCCCTTGACGGCCATTT	GTGGAGTGCTCACCCATCAA
*CKX3*	cytokinin oxidase/dehydrogenase 3	TGGGCCTCAGATTAGCAACG	CATCCTTGGTGGGTGAGCAT
*CKX4*	cytokinin dehydrogenase 4	GGAGGTGTTGTGCAAGCAAG	GAGGTAAGTGGCGTGGTCAA
*CKX5*	cytokinin dehydrogenase 5	CTGATCCGAGCGGCATACAA	GCGGTTTCCTCATCATCCCT
*CKX7*	cytokinin dehydrogenase 7-like	CTCGCATCGTCGCATTTCTC	GGATCTTCGAGGCGATTCCA
*Actin*		CTACGTCCCTGCCCTTTGTACA	ACACTTCACCGGACCATTCAA

### Analysis of *PpIPT* genes expression levels

Typically, *IPT* genes were mainly involved in CK biosynthesis, and three *PpIPT* genes were selected to analyze their expression patterns (Fig. 6, Fig. S1). The expression trend of *PpIPT* genes in GN were relatively single, and all three *PpIPT* genes were upregulated in the later stage when compared with S1, while the expression changes in LN were much more complex (Fig. 6a-c). When the two materials were compared at the same stage, *PpIPT3* was upregulated at S1 and S2 stages in LN, and downregulated at the latter three stages, while *PpIPT5* and *PpIPT8* were upregulated at the S1 stage in LN and downregulated at the later stages compared to GN (Fig. S1).

### Analysis of *PpLOG* gene expression levels

We then analyzed the expression patterns of *LOG* gene that was mainly associated with CK activation (Fig. 6, Fig. S1). Compared to S1, the *PpLOG1* gene was upregulated at S3 and S5 stages in GN, while it was only upregulated at S5 stage in LN (Fig. 6d). When comparing the two materials at the same stage, *PpLOG1* gene was only upregulated at S4 stage in LN compared to GN, and downregulated at other stages (Fig. S1).

### Analysis of *PpZOG* genes expression levels

Figure 6e-h showed *PpZOG* genes expression patterns for the GN and LN. Specifically, we analyzed the expression of four *PpZOG* genes that included two *PpZOG* genes refer to trans-zeatin O-glucosyltransferase, and two *PpcisZOG* genes refer to cis-zeatin O-glucosyltransferase, which were involved in CK inactivation (Fig. 6). The gene expression was variable for each gene across the different stages both in LN and GN (Fig. 6e-h). However, compared with the same stage of two material, the *PpZOG2* gene was only upregulated at S3 stage; the *PpZOG1* was only upregulated at S1 in LN compared to GN, but downregulated at other stages; the *PpcisZOG1* and *PpcisZOG2* were basically upregulated in LN compared to GN, except for *PpcisZOG1* at S4 (Fig. S1).

### Analysis of *PpGLU* genes expression levels

We then analyzed genes from the *GLU* genes that were mainly involved in CK re-activation (Fig. 6, Fig. S1). *PpGLU5* gene was basically opposite in GN and LN; *PpGLU26* gene was upregulated at S3 and S5 in GN, while only upregulated at S5 in LN; the expression of *PpGLU44* gene was consistent in both materials, both upregulated at S2 and downregulated in the last three stages compared to S1 (Fig. 6i-k). When compared to GN at the same stage, the expression of *PpGLU5* was upregulated at S1, S2, S4, and S5 stages in LN compared to the same stage in GN, while with the *PpGLU26* was mostly downregulated at S1 and S2, but upregulated later at S5 in LN compared to the same stage in GN (Fig. S1, Fig. 6j). And the expression of *PpGLU44* was upregulated at each stage compared to GN (Fig. S1).

### Analysis of *PpCKX*genes expression levels

The expression patterns of five *PpCKX* genes were obtained in LN and GN (Fig. 6, Fig. S1). In general, each *PpCKX* gene had different expression patterns across the different developmental stages. And the expression of five genes were diverse and complex in two materials at five developmental stages (Fig. 6l-p). At all five stages, *PpCKX2* was upregulated in LN compared to GN at the same stage. However, with *PpCKX3*, gene expression was downregulated at S1 and S2 in LN compared to GN, but upregulated at S3 and S4 stages. Additionally, with *PpCKX4*, the gene expression was upregulated at S1 stage, but downregulated at S2, S3 and S5 stages in LN compared to GN. Interestingly for *PpCKX5* (Fig. 6o) and *PpCKX7* (Fig. 6p), their gene expressions were mostly upregulated compared to GN (Fig. S1).

### Comprehensive analysis

In order to more intuitively showed the effects of nutrients, endogenous CKs and CK-related genes and on seed yield parameters, we normalized the data and made a heatmap of the integrated result (Fig. 7). According to the difference in the contents of the two materials’ panicles at different stages, nutrients were divided into three categories. And the first category including starch and NSC, was higher at S2 and S3 stages in GN, while higher at S3 and S4 stages in LN; the second category (soluble sugar) gradually increased in GN and was relatively stable in LN; the third category (soluble protein, free fatty acid and lipase activity) was higher in GN than that in LN (Fig. 7a). The changes of endogenous CKs were complex and diverse, and they were divided into four categories based on the difference in the contents of the two Kentucky bluegrass materials panicles at different stages (Fig. 7b). Among which, DHZ belonged to the first category, being lower at S1 in GN, and S2 in both materials; IPA and ZR belonged to the second category, being lower at S1, S4 and S5 in GN; DHZR belonged to the third category, being lower at S3 and S4 in GN, while higher at S3 and S4 in LN; the fourth category (IPAR and zeatin) was lower at S4 in two materials.

CKs-related genes were divided into three categories based on their expression patterns at different stages of panicle. Six genes (*PpLOG1*, *PpCKX4*, *PpIPT3*, *PpIPT5*, *PpIPT8*, and *PpZOG1*) in the first category were lower at the later stages (S1-S5) in GN, while three genes (*PpZOG2*, *PpGLU26*, and *PpCKX3*) in the second category were lower at S1 and S2 stage in GN (Fig. 7c). Interestingly, the expressions of these 10 genes in two categories in LN were complex and the regularity was not obvious. Seven genes (*PpcisZOG1*, *PpcisZOG2*, *PpGLU44*, *PpGLU5*, *PpCKX2*, *PpCKX5*, and *PpCKX7*) in the third category exhibited basically opposite trends in the two materials, which indicates that the expressions of the genes may be closely related to seed yield.

### A hypothesis cytokinin-mediated regulatory network that may produce higher seed yield parameters in GN

Taken together, our results showed that panicles in GN produced higher seed yield parameters (Fig. 2). From these relativity between CK content, CK-related gene expression and the seed yield parameters, we established a schematic diagram to illustrate their effects to increase seed production in Kentucky bluegrass (Fig. 8). We obtained three CK pathways that may produce higher seed yield parameters, which included two pathway that upregulated expression and a downregulated expression. At S1and S2 stages of panicle differentiation, *PpZOG2*, *PpGLU26*, *PpCKX3* were highly expressed. We speculated that in this pathway should include other *PpIPT* and *PpLOG* genes that were not discussed in our study, so we refer to this possible differentially expressed CK-related gene that was not discussed in this study as “potential genes”. Therefore, *PpZOG2*, *PpGLU26*, *PpCKX3* and other potential upregulated *PpIPT* and *PpLOG* genes formed the first upregulation pathway in the early stage of panicle differentiation (S1-S2), which may result in higher seed yield in Kentucky bluegrass. For the S3-S5 stages, 6 genes (*PpIPT3*, *PpIPT5*, *PpIPT8*, *PpLOG1*, *PpZOG1* and *PpCKX4*) and other potential *PpGLU* genes were highly expressed. It was important to note that these genes may be related to the DHZR content in Kentucky bluegrass. This was the second upregulated pathway in the latter stage of panicle differentiation (S3-S5), which also may result in higher seed yield in Kentucky bluegrass. Furthermore, *PpcisZOG1*, *PpcisZOG2*, *PpGLU5*,* PpGLU44*, *PpCKX2*, *PpCKX5*, *PpCKX7* were downregulated expressed and other potential *PpIPT* and *PpLOG* genes also had low expression at all five stages of panicle development. These three pathways co-regulated endogenous CKs content affected the seed yield parameters in Kentucky bluegrass. Overall, our research results indicated a correlation between CK contents and seed yield parameters, so differential expression of CK-related genes may increase the seed yield in *Poa pratensis*.

## Discussion

Young panicle differentiation marks the transformation of vegetative growth to reproductive growth that produces reproductive organs in Kentucky bluegrass. Whether the young panicle can develop well or not directly determines the seed yield and later economic benefits [27]. Therefore, the end of spikelet initiation is usually identified during grass management because it can be used to predict yield success [28]. Previous study also indicated that wheat (*Triticum aestivum*) early spike development is controlled by a small subset of genes that regulate CK signaling [29]. In order to confirm whether CKs regulate seed yield and quality for panicle differentiation, we investigated seed yield parameters with different nutrients, CK contents, and CK-related gene expression changes in two wild Kentucky bluegrass in Gansu with different seed yield performance. Together, our research findings provided new insights and strategies for the cultivation of seed high-yield and high-quality new varieties of Kentucky bluegrass, which may be achieved by disrupting endogenous CKs by manipulating CK-related genes during panicle differentiation.

### Significance and function of the panicle differentiation process

Previous study have explored the development of grass spikelets, flowers, and caryopse, and their homologies in floral parts, but the different stages of inflorescence not been investigated, especially at the early stages [30]. Of course, in the main cereal crops, young spike/panicle differentiation serves as the basis for grain yield, and there have been relevant reports [31, 32]. However, forages and grasses, like Kentucky bluegrass, have also not been explored in-depth during these different inflorescence stages. Previously, Li et al. [33] suggested that panicle development was an important factor that determined spikelet and floret number, but it did not affect seed. However, the number of spikelets and florets constituted the SSR, which did affect seed yield, so the current view was that the panicle development was the foundation of seed yield, and its quality directly affected the parameters of seed yield in Poaceae plants [34]. In our study, the panicle differentiation of Kentucky bluegrass was divided into five stages in the two materials based on the external morphological changes (Fig. 3). Meanwhile, the different phenological stages of panicle differentiation were different (Table 2), so targeted control measures can be taken to increase the seed yield and quality at different stages of young panicle differentiation in Kentucky bluegrass.

### Association of nutrient content with seed yield parameters during panicle differentiation

Sugars, proteins and lipids are important regulators of plant growth, development and gene expression, and they are also essential for energy resources, structural substances and plant survival. Sugar can act as a signaling molecule, in combination with phytohormones, to regulate plant organ development through complex signal transduction mechanisms [35]. Panicle development was also related to carbohydrate accumulation, and insufficient supplies can lead to pollen abortion [7, 36]. These carbohydrates were usually classified into structural carbohydrates (SCs) and NSCs based on their presence in plants [37]. Typically, NSCs acted as temporary storage reservoirs for carbohydrates that are overproduced during photosynthesis, and included soluble sugar and starch [38]. In our study, our results showed that, in GN, soluble sugar content was gradually increased with the developmental stage, starch peaked earlier, NSC was higher at S2 and S3, and significantly decreased at the S4 stage, suggesting that these changes may be a signature of sugar metabolism that produced higher seed yield parameters in Kentucky bluegrass (Fig. 4a, b, c). With proteins and fatty acids, the miR1432-OsACOT module played an important role in seed filling by involving in biosynthesis of medium-chain fatty acids [39]. In our data, we found that the soluble protein content in panicles from GN was substantially higher than that in LN at S1, S2 and S5 stages, but there was no difference at S3 and S4 stages (Fig. 4d). We also found a similar trend with the free fatty acid content and lipase activity in GN, among which free fatty acid was significantly higher than that in LN at S5, and lipase activity was higher than that in LN at S4 and S5 (Fig. 4e, f).

Altogether, our results suggested that the nutrient content during panicle differentiation were different in two materials with different seed yield parameters, so we speculated that seed yield parameters were associated with nutrient contents. Specifically, our correlation analysis showed that NSC and SEN, as well as SSR and lipase activity were positively correlated at S3 in both GN and LN (Table 3), which suggested that SEN and SSR improvements may be achieved by strengthening the NSC content and lipase activity at the S3 stage during seed production, respectively. However, this requires further experiment validation, and exogenous application may be a very good method that will be considered in future work.

### Association of endogenous CKs content during panicle differentiation with seed yield parameters

With endogenous CKs, Chen et al. [40] demonstrated that there was a significant positive correlation between seed weight, filling rate harvest index and CK content. Additionally, previous study on barley revealed a significant increase in CKs during seed filling, especially zeatin, and they found greater CK concentrations in the cultivar that produced the greatest number of seeds per spike and was also the highest yielding cultivar [16]. In other crops like wheat, Zheng et al. [41] also demonstrated that the foliar spraying of 6-BA (6-benzyladenine, it is the first artificially synthesized CK) increased the seed number and SSR of fertile florets by suppressing their abortion at each spikelet position on each spike, and concluded that the application of 6-BA improved seed yield. Yang et al. [42] found that the levels of multiple CKs were markedly elevated in young mutant panicles that produced more spikelets. In our study, the results showed that CKs were in dynamic equilibrium during the different panicle differentiation stages. Interestingly, the zeatin content in GN was significantly higher than that in LN at S1-S3 stages (Fig. 5), so we concluded that higher zeatin content in the panicle was associated with increased seed number and size. At the early seed formation stage, ZR content could effectively regulate flower development and spikelet number, and higher ZR content could increase yield sink capacity [43]. In this work, our correlation analysis suggested that PBNSN, TGW and ZR content at the S1 and S2 stages in two materials were significantly and positively correlated, which indicated that PBNSN and TGW may be improved by increasing ZR content (Table 3). Additionally, our comprehensive analysis suggested high DHZR content during the S3-S5 stages facilitated seed growth during panicle development, and DHZR belonged to the third category, being lower at S3 and S4 in GN, while higher at S3 and S4 in LN, which seemed likely that the DHZR content was inversely proportional to the seed yield (Figs. 7 and 8). On the other hand, it was noteworthy that out of the six CK concentrations quantified in this study, five (zeatin, ZR, DHZR, IPA and IPAR) kinds were higher in the GN than those in LN at S3 (Fig. 5). CK was well known to maintain meristematic activity and regulate meristem size, and S3 represented the spikelet and floret primordium differentiation stage. In addition, based on the phenotypic quantification (Table 3), both spikelet and floret numbers were greater in GN than those in LN, and we also found that GN had more meristems compared to LN. So, we concluded that spikelet and floret primordium differentiation stage with higher endogenous CK content can promote more spikelets and florets, which may subsequently increase the seed yield in Kentucky bluegrass.

### Regulation of CKs-related genes during panicle differentiation and their association with seed yield parameters

There were several previous studies regarding the regulation of seed yield and quality by CKs [44–46]. For example, Ashikari et al. [47] previously found that there was a positive relationship between the number of seeds, branches, and CK content in rice. Others also suggested that the *IPT*, *LOG*, *ZOG*, *GLU*, and *CKX* genes controlled CK content [48]. Typically, CK biosynthesis was regulated by *IPT* genes, which catalyzed the initial and rate-limiting steps [49]. Previous study have reported the relative expression levels of *TaIPT2* in three high yielding cultivars were approximately double those of the two low yielding cultivars [48], which was generally consistent with previous results of wheat [50]. Generally high expression of two CK biosynthesis genes (*TaIPT2* and *TaIPT8*) during seed development indicated that denovo CK biosynthesis had a major role in wheat kernel development [48]. In our work, we found that the expression patterns of *PpIPT3*, *PpIPT5* and *PpIPT8* were largely consistent in both materials, and that *PpIPT3* was downregulated during the S3-S5 stages, while *PpIPT5* and *PpIPT8* were downregulated at the last S2-S5 stages (Fig. S1). Combined our seed trait analysis, our results demonstrated that higher expression of the *PpIPT* genes during panicle differentiation were beneficial to increase seed yield parameters (Fig. 7). While *IPT* was the limiting step for CK biosynthesis, attempted at genetic modifications that aimed to increase CK biosynthesis have been challenging to control [51]. Furthermore, while examples of ectopic *IPT* expression have increased traits related to seed yield, there have been no natural variants that are linked to high yield in crop species [52]. Therefore, further validation of the correlation between *PpIPT* expression levels and seed yield is needed through gene editing techniques.

Additionally, the *LOG* genes encoded a novel CK-activating enzymes that worked in the final step of bioactive CK synthesis [53, 54]. Members of the *LOG* gene family numbers were differentially expressed in various tissues during plant development, and had overlapping but differentiated functions in growth and development [55], such as rice *LOGL6* regulated rice awn and grain production [56]. Additionally, Wang et al. [57] reported herein that constitutively overexpression of *OsLOGL5* conferred short roots, less tillers, and semi-dwarf phenotypes, whereas mutations of *OsLOGL5* produced normal rice plants with significantly increased grain yield under well-watered, drought, normal nitrogen, and low nitrogen field conditions, which indicated that *OsLOGL5* can play an important role in grain yield stability under various field conditions. Notably, we found *PpLOG1* was downregulated in four stages in LN, except for S4 stage (Fig. S1), which indicated that the over expression of *PpLOG1* could help to increase seed development (Fig. 8). With CKs, the *ZOG* and *GLU* gene family were also mainly involved in CK inactivation and re-activation, respectively [18]. It has been shown that the highest yield cultivar presented the lowest expression levels of *TacisZOG1*, *TacisZOG2a* and *TacisZOG2b*, and the expression of *TacisZOG2a* was generally lower in all three high yield cultivars compared to the two low yield wheat [48]. Moreover, one of the lower yielding cultivars had a higher level of *Glu1-1* gene expression than those of all higher yielding wheats [48]. Interestingly, we found *PpcisZOG1*, *PpGLU5* and *PpGLU44* were all downregulated in GN (Fig. S1), which revealed that the CK activity varies greatly in GN that had high seed yield parameters. Together, these results demonstrated that these genes acted as lowly-expressed pathway members that regulate seed growth (Figs. 7 and 8). Furthermore, the expression characteristics of *PpZOG2* and *PpGLU26* changed through the different developmental stage, and were highly expressed during the first two S1-S2 stages to promote seed yield. Altogether, these changes in *LOG*, *ZOG* and *GLU* gene expression may be manipulated to assess whether seed production and yield can be improved in Kentucky bluegrass.

Along with the other CK-related genes, the *CKX* genes catalyzed the irreversible degradation of CKs and modulated their cellular levels, which were the most well-studied CK-associated genes that regulated seed yield [58, 59], and their disruption led to higher seed number [60]. Sextuple *ckx3 ckx5* mutants rape showed that weight of seeds harvested from the main stem of plants grown in the greenhouse or in the field was increased by 20–32% [25]. Consistently, it was shown that an increased root to shoot transport of CK positively influenced yield in rice [61]. For example, the ERECTA pathway controlled meristem size by inducing *OsCKX2* to reduce CK levels [25, 62]. *CKX* genes associated with yield-related traits had also been identified in barley [63] and wheat [64]. These works all demonstrated the potential to achieve yield enhancement in crop plants by modulating the CK status through mutagenesis of specific *CKX* genes, and in cereals the CK status and its regulation by *CKX*s was a well-established determinant of seed yield. In this work, we found that five *PpCKX* genes could be divided into three categories based on expression patterns. The first category included the *PpCKX2*, *PpCKX5* and *PpCKX7* genes, which were downregulated in GN at each stage compared to LN (Fig. 7 and Fig. S1). These data indicated the lower expression of the first category such as *PpCKX* genes were associated with improving seed yield (Fig. 8). Additionally, the expression of the *PpCKX3* and *PpCKX4* genes were related to different developmental stages. We found that *PpCKX3* was involved in high expression during the first S1-S2 stages, and *PpCKX4* was involved in the S3-S5 stages to increase seed yield parameters (Fig. 8). The results suggested the regulation of crop seed yield by *CKX* genes had period specificity and may also have tissue specificity, which may be important works worth further exploration in the future.

Taken together, we found that changes in CK content were associated with seed yield in Kentucky bluegrass, and that CKs levels can be determined by CK-related genes, such as the *IPT* genes for CK biosynthesis, the *PpLOG* genes for activation, the *PpZOG* genes for inactivation, the *PpGLU* genes for re-activation, and *PpCKX* genes for degradation [65, 66]. Thus, CKs-related genes manipulation may be used to alter CK content during panicle differentiation using modern genome editing tools to improve seed yield for *P. pratensis*. Undoubtedly, these inferences require experimental verification. For instance, it could be determined whether seed yield parameters of Kentucky bluegrass can be improved by the over-expression of the *PpIPT* and *PpLOG* genes such as the *PpITP3*, *PpITP5*, *PpITP8* and *PpLOG1* respectively, or silencing and editing *CKX* genes such as the *PpCKX2*, *PpCKX5* and *PpCKX7* genes explored in this work. Ultimately, these CK-related genes and other CKs could be investigated in other crops to improve their seed yield and economic potential.

## Conclusion

In this work, we found that panicle differentiation and development were the basis for seed yield and quality, and that CK played a critical role in regulating spikelet and floret number, which directly affected seed yield. From our data, we discovered an indirect link between seed yield and CKs, and showed that higher zeatin content during panicle differentiation was associated with an increase in TGW in Kentucky bluegrass, as well as SSR and lipase activity at the S3 stage showed a significant positive correlation. Additionally, Kentucky bluegrass materials with higher seed yields contained higher endogenous cytokinins during the young panicle differentiation stage. Especially during the spikelet and flower primordia differentiation stage, all of zeatin, ZR, DHZR, IPA, and IPAR contents were significantly higher in high-yield materials than those in low-yield materials. Furthermore, the *PpITP3*, *PpITP5*, *PpITP8* and *PpLOG1* were high expressed in Kentucky bluegrass materials with higher seed yields, while the *PpCKX2*, *PpCKX5* and *PpCKX7* were low expressed, so modern genome editing tools may be employed to target and manipulate CK levels to increase seed yield to maintain or improve crop-yield of Kentucky bluegrass in the future during young panicle differentiation. Ultimately, these relationships we defined between nutrients, CKs, and CK-related genes can be used in the future to manipulate seed yield and improve crop-yields for a variety of Kentucky bluegrass.

## Materials and methods

### Experimental materials and measurement of the seed yield parameters

In the early stage, we collected wild germplasm materials of Kentucky bluegrass from Gannan (GN) and Longnan (LN) in Gansu, China [3]. We have permission to collect plant material. Te voucher specimens, GN (HNWP 0285644) was identified by Kun Liu and its sheet was deposited in the herbaria HNWP (https://www.cvh.ac.cn/spms/detail.php? id=ec618bf6), and LN (PE 00977532) was identified by Quanxi Li and its sheet was deposited in the herbaria PE (https://www.cvh.ac.cn/spms/detail.php? id=efb4e16e). They could be also searched on the Chinese Virtual Herbarium (https://www.cvh.ac.cn/spms/list.php?) and code (00977569, 019104175 and 0089157). In a previous study, we learned that seed yield-related traits differed significantly between GN and LN [26]. In late April 2020, GN and LN samples were planted on individual plants at the lawn training base of Gansu Agricultural University, with 50 plants per material.

We randomly selected 30 reproductive branches to count the seed yield traits that directly affected by panicle differentiation. The characteristic and their abbreviation of these seed yield parameters were shown in Table 1. In total, 50 seeds were selected to measure seed length, width, length/width, perimater, and area using PhenoAI (version: 2.1.15, AgriBrain Co., Ltd, Nanjing, China). The inflorescence structure of wild germplasm materials of Kentucky bluegrass were shown in Fig. 1.

### Dynamic process observation of panicle development

From mid-February 2022, the growth of Kentucky bluegrass was recorded. Total of 30 single plants with consistent growth, and 1 branch from each plant were randomly selected. A total of 30 branches were used to observe the process of young spike differentiation, with samples taken every 3 days. A microscope (Version: Discovery.V12, Carl Zeiss, Germany) was used to observe and photograph changes in growth points.

According to the dynamic process observation, the panicles from LN were sampled on March 3, March 12, March 21, April 2, and April 15, and panicles from GN were sampled on March 15, March 24, April 2, April 14, and April 25, respectively. There five samples were labeled as S1, S2, S3, S4 and S5, respectively. The collected panicles were immediately and rapidly frozen in liquid nitrogen and stored at -80 °C for nutrient analysis, endogenous CKs contents determination and RNA extraction. Three biological replicates were set at each time point, and each sample was taken from a mixed collection of at least five plants.

### Determination of nutrients during panicle differentiation in wild Kentucky bluegrass

Using the two collected materials from 5 developmental stages of young panicles, three biological replicates were conducted to determine the nutrient content of a total of 30 samples. The protein content was determined by Bicinchoninicacid (BCA) analysis using the Kit (Solarbio Co., Ltd., Beijing, China). According to the manufacturer’s instructions, 0.1 g sample of fresh panicles was taken and ground thoroughly, and 1 mL of distilled water was then added to the sample. Subsequent steps were performed following the manufacturer’s instructions, and protein were detected at and 562 nm.

The soluble sugar and starch content were measured using the Anthrone Colorimetry Kit (Suzhou Mengxi Biotechnology Co., Ltd., Suzhou, China). Methods were repeated as previously stated, and subsequent steps were performed, following the manufacturer’s instructions. The soluble sugar and starch were detected at 620 nm at the same absorbance. More than 90% of the NSC in plants were soluble sugars and starch, so we expressed the NSC content as their sum total.

The content of free fatty acid was determined by Kit (Suzhou Mengxi Biotechnology Co., Ltd., Suzhou, China), and 0.1 g sample of fresh panicles was mashed and subsequent steps were performed, following the manufacturer’s instructions. The free fatty acid was detected at 715 nm.

The lipase activity was determined by Kit (Suzhou Mengxi Biotechnology Co., Ltd., Suzhou, China). In ice-cold mortar, 0.1 g sample of fresh panicles was ground thoroughly and subsequent steps were performed following the manufacturer’s instructions. The lipase activity was detected at 405 nm.

### Detection of endogenous CKs contents

ELISA method was performed to detect CKs contents in panicles, including zeatin, dihydrozeatin (DHZ), isopentenyl adenosine (IPA) as well as their ribosides (ZR, DHZR, IPAR). CKs were extracted using the method described by Lu et al. [67] with some modifications. A 0.1 g sample of fresh panicles was taken and ground thoroughly under liquid nitrogen, then dissolved in a 0.5 mL-extract solution that contained methanol, water, and formic acid (v: v:v = 15:4:1). After 10 min of extraction, the supernatant was obtained by centrifugation (5 min at 12,000 rpm). The extraction and centrifugation steps were repeated twice. The all supernatants that contain all of the obtained supernatants mentioned above were pooled together and concentrated to dry at 40 °C using a vacuum decompression concentrator. The extracts were then resuspended with 100 µL of an 80% methanol-water solution and sonicated for 1 min, followed by filtration through a 0.22-micron polytetrafluoroethylene membrane. Six types of CK contents were detected using ELISA Kits following the manufacturer’s instructions (Shanghai Zhili Biotechnology Co., Ltd., Shanghai, China).

### Quantitative fluorescence PCR (q-PCR) of CK-related genes

Based on the inflorescence transcriptome of Kentucky bluegrass [3], we screened for and obtained 16 CK-related genes from a BLAST search (National Center for Biotechnology Information (NCBI). These CK-related genes included three *PpIPT* genes, a *PpLOG* gene, four *PpZOG* genes, three *PpGLU* genes, and five *PpCKX* genes, the original sequencing reads have been submitted to the Sequence Read Archive (SRA) at NCBI (Accession Number: PRJNA680673). Specific PCR primers were designed using the NCBI online website Primer-BLAST (http://www.ncbi.nlm.nih.gov/tools/primer-blast). The relevant information and sequences of these genes were included in the Table S5, and the primer sequence of 16 genes were listed in Table 4. The total RNA was extracted from the 30 samples using an RNA easy Plant Mini Kit according to the manufacturer’s instructions (Qiangen, Hilden, Germany). The concentrations of RNA were then detected using an Ultra Micro UV Spectrophotometer (Beijing Dinghaoyuan Biotechnology Co., Ltd., Beijing, China). The total RNA was then reverse-transcribed into cDNA using a Prime Script RT reagent Kit with a gDNA Eraser (Perfect Real Time) (TaKaRa, Japan) and diluted 20 times as template. Prior to conducting reverse transcription experiments, RNA samples were subjected to DNase treatment to remove DNA contamination from them. The qRT-PCR was performed with StepOnePlus™ Real-Time PCR System (ABI) using SYBR premix Ex Taq (Takara, Japan). Triplicate qRT-PCR of each sample was performed, with each biological repetition being the mean of 3 technical repetition in Fig. 6. The reaction volume was 20 µL and included 10 µL of 2 × SuperReal PreMix Plus, 3 µL of ddH_2_O, 5 µL of cDNA template, and 2 µL of forward and reverse primer. The total reaction conditions included a pre-denaturation step at 95 °C for 15 min, 40 cycles of PCR amplification (denaturation at 95 °C for 10 s and annealing at 58 °C for 30 s). Specific methods referred to the published literature [1, 3]. *Actin* was used as an internal reference gene, and its Ct values of 10 samples were in the Table S6 the relative expression levels of each genes was calculated by 2^−∆∆Ct^ [68].

### Data analysis and statistics

Data were analyzed using independent sample *t*-tests and One-way ANOVA with SPSS software (version 19.0 IBM Corp., Armonk, NY, USA) in Windows. GraphPad Prism software (version 8.0.2. San Diego, CA, USA) was used for drawing. And heatmap was drawn using OmicShare tools, a free online platform for data analysis (http://www.omicshare.com/tools).

### Electronic supplementary material

Below is the link to the electronic supplementary material.

Supplementary Material 1

Supplementary Material 2

Supplementary Material 3

Supplementary Material 4

Supplementary Material 5

Supplementary Material 6

Supplementary Material 7

## Data Availability

All relevant files are included in this article and its supplementary files.

## Abbreviations

CK: Cytokinin
IPT: Isopentenyl Transferases
LOG: Lonely Guy
ZOG: O-glucosyltransferases
CisZOG: Cis-O-Glucosyltransferases
GLU: β-Glucosidases
CKX: Cytokinin Oxidase/Dehydrogenase
SC: Structural Carbohydrates
NSC: Nonstructured Carbohydrates
ZR: Zeatin-Riboside
DHZ: Dihydrozeatin
DHZR: Dihydrozeatin Riboside
IPA: Isopentenyl Adenosine
IPAR: Isopentenyl adenosine riboside
6-BA: 6-benzyladenine
