# Supplementary material for: Seed yield as a function of cytokinin-regulated gene expression in wild Kentucky bluegrass (Poa pratensis)
Source: BMC Plant Biol. 2024 Jul 20;24:691. doi: 10.1186/s12870-024-05421-w (PMC11265001; doi:10.1186/s12870-024-05421-w)
Supplement: Supplementary file 7 — Supplementary Material 7 [file 12870_2024_5421_MOESM7_ESM.docx]

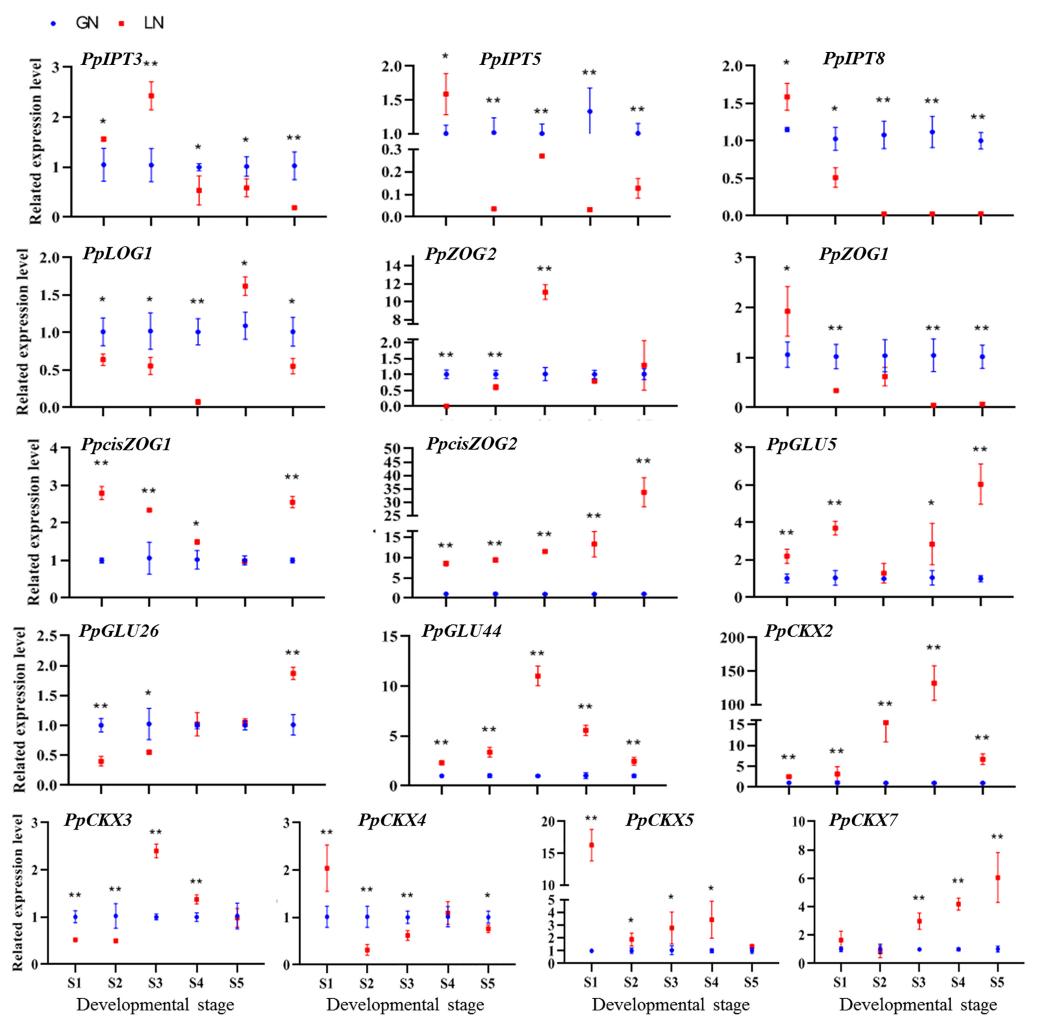


Fig. S1 The relative expression levels of CK-related genes in LN compared to GN at different developmental stages of panicle differentiation.
